# Treatment with the Topical Antimicrobial Peptide Omiganan in Mild-to-Moderate Facial Seborrheic Dermatitis versus Ketoconazole and Placebo: Results of a Randomized Controlled Proof-of-Concept Trial

**DOI:** 10.3390/ijms241814315

**Published:** 2023-09-20

**Authors:** Jannik Rousel, Mahdi Saghari, Lisa Pagan, Andreea Nădăban, Tom Gambrah, Bart Theelen, Marieke L. de Kam, Jorine Haakman, Hein E. C. van der Wall, Gary L. Feiss, Tessa Niemeyer-van der Kolk, Jacobus Burggraaf, Joke A. Bouwstra, Robert Rissmann, Martijn B. A. van Doorn

**Affiliations:** 1 Centre for Human Drug Research, 2333 CL Leiden, The Netherlands; 2 Leiden Academic Centre for Drug Research, Leiden University, 2333 CC Leiden, The Netherlands; 3 Leiden University Medical Center, Leiden University, 2333 ZA Leiden, The Netherlands; 4 Westerdijk Fungal Biodiversity Institute, 3508 AD Utrecht, The Netherlands; 5 Cutanea Life Sciences, Wayne, PA 19087, USA; 6 Department of Dermatology, Erasmus Medical Centre, 3015 GD Rotterdam, The Netherlands

**Keywords:** seborrheic dermatitis, omiganan, ketoconazole, *Malassezia*, *Staphylococcus*, skin barrier, multimodal

## Abstract

Facial seborrheic dermatitis (SD) is an inflammatory skin disease characterized by erythematous and scaly lesions on the skin with high sebaceous gland activity. The yeast *Malassezia* is regarded as a key pathogenic driver in this disease, but increased *Staphylococcus* abundances and barrier dysfunction are implicated as well. Here, we evaluated the antimicrobial peptide omiganan as a treatment for SD since it has shown both antifungal and antibacterial activity. A randomized, patient- and evaluator-blinded trial was performed comparing the four-week, twice daily topical administration of omiganan 1.75%, the comparator ketoconazole 2.00%, and placebo in patients with mild-to-moderate facial SD. Safety was monitored, and efficacy was determined by clinical scoring complemented with imaging. Microbial profiling was performed, and barrier integrity was assessed by trans-epidermal water loss and ceramide lipidomics. Omiganan was safe and well tolerated but did not result in a significant clinical improvement of SD, nor did it affect other biomarkers, compared to the placebo. Ketoconazole significantly reduced the disease severity compared to the placebo, with reduced *Malassezia* abundances, increased microbial diversity, restored skin barrier function, and decreased short-chain ceramide Cer[NSc34]. No significant decreases in *Staphylococcus* abundances were observed compared to the placebo. Omiganan is well tolerated but not efficacious in the treatment of facial SD. Previously established antimicrobial and antifungal properties of omiganan could not be demonstrated. Our multimodal characterization of the response to ketoconazole has reaffirmed previous insights into its mechanism of action.

## 1. Introduction

Seborrheic dermatitis (SD) is a chronic inflammatory skin disease characterized by erythematous, scaling, and indurated papules and plaques on the face, scalp, and upper chest that affects up to 3% of the general population. While aberrant immunological responses are integral to its pathophysiology, these appear to be induced or exacerbated by multiple co-factors, such as an impaired barrier function, environmental factors, and microbial disturbances [1]. 

Microbial involvement is characterized by the commensal yeast *Malassezia* that acts as a key pathogenic driver in SD [2]. *Malassezia* thrives on the sebum-rich areas of the face and scalp where its lipase activity disturbs the skin barrier, facilitating the penetration of exogenous compounds including *Malassezia*’s own pro-inflammatory metabolites [3,4]. Besides the clear mycobial involvement, SD is associated with microbial dysbiosis, exemplified by increased abundances of *Staphylococcus* [5]. The potential contribution of *Staphylococcus* to the pathophysiology of SD is less clear but may be highly relevant in view of the prominent role of *S. aureus* in atopic dermatitis, where it is considered a potential target for treatment [6,7]. 

While it is hypothesized that the presence of *Malassezia* only facilitates inflammation instead of being a causative factor in SD development, antifungal treatment with imidazole derivatives such as ketoconazole is an established first-line topical treatment for mild SD [8]. Since standalone antifungal therapy might not be sufficiently effective, combination treatment with topical corticosteroids is often required. However, corticosteroid use is constrained by several well-known side effects upon long-term exposure [9]. The absence of efficacious and safe treatment modalities highlights the need for novel therapies as SD continues to impact the quality of life, especially when located on the face [10]. 

Based on the high degree of microbial involvement, targeting both the fungal and bacterial microbiome through antimicrobial peptides (AMPs) is hypothesized to be a promising therapeutic option. AMPs are part of the skin’s innate immune response and are upregulated upon injury or cutaneous inflammation, providing an immediate host response against pathogens on the skin [11]. 

Omiganan is an AMP that is analogous to indolicidin, a bovine member of the cathelicidin family. Its naturally occurring human counterpart LL-37 and derivatives have shown to be effective against selected *Malassezia* and *Staphylococcus* strains [12,13,14,15]. Omiganan itself has shown antibacterial and antifungal activity throughout a range of preclinical and clinical studies [16,17,18,19,20,21,22], with a favorable safety profile and microbial target engagement in doses from 1% to 2.5% [20,21,22,23,24]. 

Ultimately, the broader spectrum activity of AMPs such as omiganan could make these compounds a valuable therapeutic option for patients with SD that might render combination with corticosteroids unnecessary. In this study, we investigated the clinical efficacy, safety, tolerability, microbiological, and pharmacodynamic effects of omiganan 1.75% versus ketoconazole and a placebo in patients with mild-to-moderate facial SD. 

## 2. Results

A total of 115 patients were screened, of which 37 were enrolled into the study and 36 successfully completed the study (Supplementary Figure S2). One patient randomized to ketoconazole was replaced due to a history of atopic dermatitis that remained undeclared at screening until a flare-up during the study, which was in violation of the study protocol. Baseline characteristics and exposure were comparable between treatment groups (Table 1). A total of 12 treatment emergent adverse events were reported by 11 subjects. Application site reactions in the omiganan and placebo groups occurred in two subjects. Other adverse events were not considered related to the study drug. 

### 2.1. Omiganan Does Not Show Clinical Improvement Compared to Placebo

**Figure 1:**
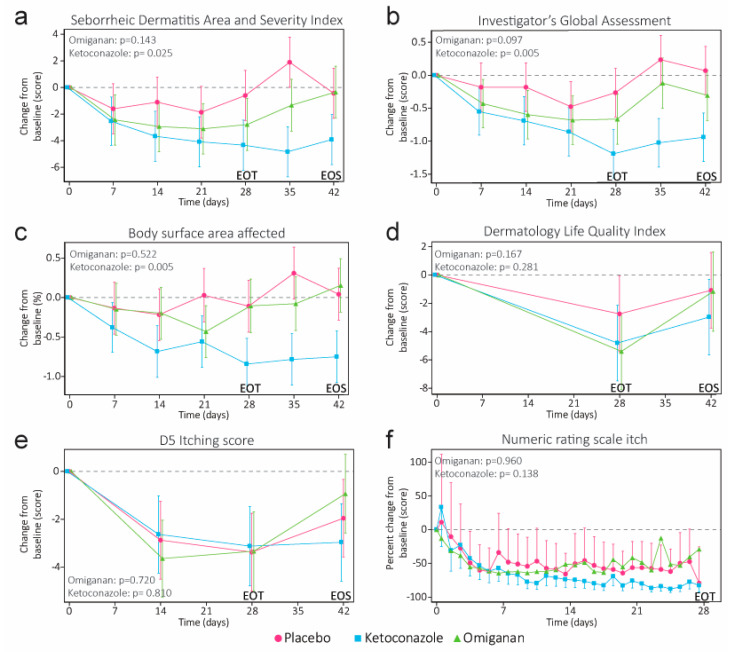
(**a**) Clinical scoring by Seborrheic Dermatitis Area and Severity Index, (**b**) Investigator’s Global Assessment, and (**c**) percentage of body surface area affected. Patient-reported outcomes by (**d**) the Dermatology Life Quality Index and (**e**) the 5-D Itch questionnaire were supplemented with (**f**) twice daily reporting of the numeric rating scale (NRS) for the severity of itch (NRS-itch). For clarity, only the evening NRS-itch has been graphed. Note that the NRS-itch was log-transformed to allow for statistical analysis and, therefore, is shown as percent change. *p*-values denote significance at the end of treatment (EOT). End of study (EOS) denotes the final follow-up visit.

Disease severity did not significantly decrease in the omiganan group compared to the placebo, as scored by SDASI (−1.5, CI −3.6 to 0.5, *p* = 0.1429), IGA (−0.3, CI −0.7 to 0.1, *p* = 0.0971), and %BSA (−0.11, CI −0.47 to 0.24, *p* = 0.5220) at EOT (Figure 1a–c). In contrast, disease severity significantly decreased after treatment with ketoconazole (SDASI: −2.4, 95% CI −4.4 to −0.3, *p* = 0.0247; IGA: −0.5, 95% CI −0.9 to −0.2, *p* = 0.0054; %BSA: −0.51, 95% CI −0.86 to −0.16, *p* = 0.0052). Patients completed questionnaires during their visit and the twice daily NRS-itch at home. Quality of life determined by DLQI was ameliorated in both the omiganan (−2.6, 95% CI −6.4 to −1.1, *p* = 0.1671) and ketoconazole (−2.1, 95% CI −5.8 to −1.7, *p* = 0.2811) treatment groups compared to the placebo at EOT but did not reach statistical significance (Figure 1d). The severity of itch in all three groups was similar (Figure 1e,f), as the 5-D Itch questionnaire, completed during the study visit, and the twice daily NRS-itch, completed by patients at home, showed a similar time-course upon starting either treatment.

### 2.2. Neither Omiganan Nor Ketoconazole Show Effects on Clinical Imaging Compared to Placebo

**Figure 2:**
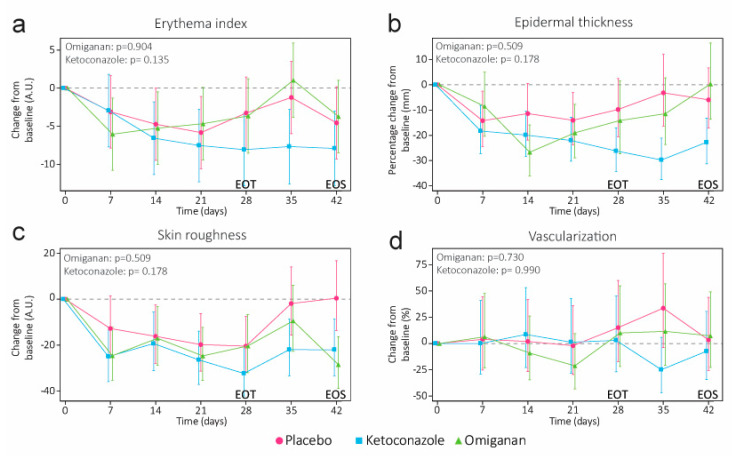
(**a**) Degree of erythema as determined by the erythema index through standardized photography. (**b**) Optical coherence tomography was used to measure the epidermal thickness, (**c**) superficial skin roughness, and (**d**) degree of vascularization. Epidermal thickness could not be determined in all scans due to the high epidermal disorganization (see the Supplementary Materials). *p*-values denote significance at the end of treatment (EOT). End of study (EOS) denotes the final follow-up visit.

Erythema was comparable to the placebo in the omiganan group (−0.1682, 95% CI −2.9839 to 2.6475, *p* = 0.9044) and non-significantly reduced in the ketoconazole group (−2.0531, 95% CI −4.7713 to 0.6652, *p* = 0.1346) at EOT (Figure 2a). No statistically significant differences were observed for epidermal thickness, roughness, or vascularization over the course of the treatment period. Compared to the placebo, skin roughness was slightly reduced by −5.3% for omiganan (95% CI −19.8% to 11.8%, *p* = 0.5090) and −10.4% for ketoconazole (95% CI −23.7% to 5.3%, *p* = 0.1778). Changes in blood flow were similar for both omiganan (−6.6%, 95% CI −37.0% to 38.6%, *p* = 0.7279) and ketoconazole (0.2%, 95% CI −33.0% to 50.0%, *p* = 0.9901) at EOT. Epidermal thickness was reduced but was not significantly lower compared to the placebo at EOT (omiganan: −5.3%, 95% CI −19.8% to 11.8%, *p* = 0.5090; ketoconazole: −10.4%, 95% CI −23.7% to 5.3%, *p* = 0.1778). For ketoconazole only, this reduction culminated in a significant 14.9% (95% CI −25.2% to −3.1%, *p* = 0.0185) reduction in epidermal thickness two weeks after the final administration. 

### 2.3. Skin Barrier Function Improves upon Treatment with Ketoconazole

**Figure 3:**
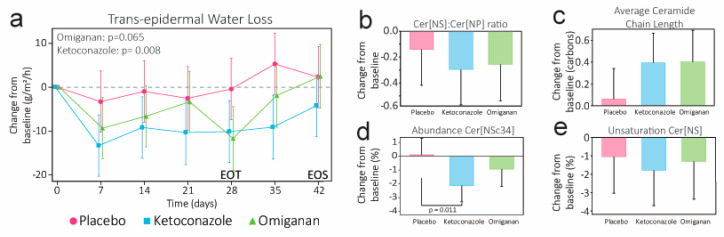
(**a**) Assessment of the cutaneous barrier function over time by trans-epidermal water loss measurements. Corresponding molecular ceramide biomarkers for barrier integrity were derived from the total stratum corneum ceramide profile and show the change in: (**b**) the ratio of the relative abundance of Cer[NS] and Cer[NP], (**c**) the carbon chain length of ceramides, (**d**) the abundance of Cer[NSc34] in total Cer[NS], and (**e**) the fraction of unsaturated ceramides in total Cer[NS]. *p*-values denote significance at the end of treatment (EOT). End of study (EOS) denotes the final follow-up visit.

TEWL was not significantly reduced compared to the placebo at EOT for omiganan (−5.935, 95% CI −12.265 to 0.395, *p* = 0.0654), but was significantly lower for ketoconazole (−8.932, 95% CI −15.367 to −2.497, *p* = 0.0076) (Figure 3). Molecular features correlating to barrier integrity showed improvements. Skewing of the ceramide subclass synthesis could be visualized by the Cer[NS]:Cer[NP] ratio. This ratio was reduced compared to the placebo by both omiganan (−0.1155, 95% CI −0.5114 to 0.2805, *p* = 0.5563) and ketoconazole (0.1549, 95% CI −0.5529 to 0.2430, *p* = 0.4332), but it did not reach significance. Similarly, the degree of unsaturation within Cer[NS] was reduced (omiganan: −0.2522, 95% CI −3.1697 to 26653, *p* = 0.8612; ketoconazole: −0.7301, 95% CI −3.5100 to 2.0498, *p* = 0.5960) and the ceramide elongation was increased (omiganan: 0.340, 95% CI −0.073 to 0.752, *p* = 0.103; ketoconazole: 0.332, 95% CI −0.061 to 0.726, *p* = 0.0951) for both treatments compared to the placebo, without reaching statistical significance. However, the abundance of Cer[NSc34] was significantly lower compared to the placebo in the ketoconazole group (−2.2344%, 95% CI −3.9182 to −0.5505, *p* = 0.0110), but not in the omiganan group (−1.0454%, 95% CI −2.7724 to 0.6816, *p* = 0.2263). Sebum measurements did not show any differences and were troubled by high standard deviations (Figure S3).

### 2.4. Ketoconazole but Not Omiganan Reduces Malassezia Abundances

**Figure 4:**
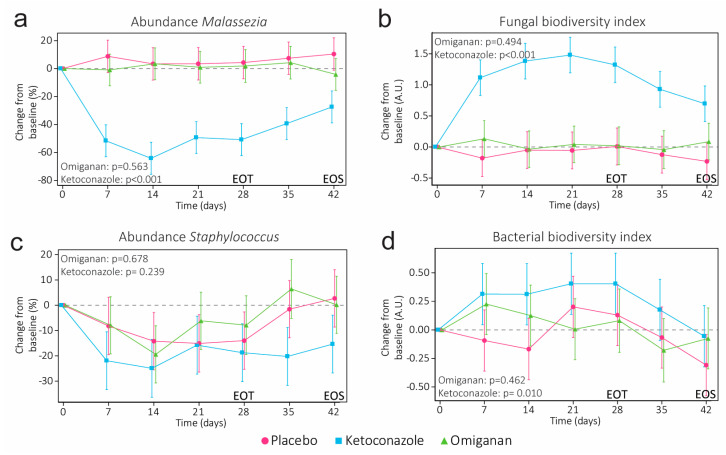
The change in relative abundance of (**a**) *Malassezia* and (**c**) *Staphylococcus* and the corresponding microbial Shannon diversity indexes for the (**b**) fungal and (**d**) bacterial microbiome. Abundances and diversity indexes are condensed from the full microbial profiles, as shown in the Supplementary Materials. *p*-values denote significance at the end of treatment (EOT). End of study (EOS) denotes the final follow-up visit.

Treatment with omiganan did not result in significant mycobial changes, with a −3.662% decrease in *Malassezia* (95% CI −16.366 to 9.042, *p* = 0.5630) and a 0.1086 increase in the Shannon diversity index (95% CI −0.2097 to −0.4270, *p* = 0.4942) compared to the placebo (Figure 4a,b). The impact of ketoconazole on the mycobiome was considerable, with significant changes compared to the placebo in both the abundance of *Malassezia* (−59.018%, 95% CI −71.952 to −46.084, *p* < 0.0001) and the fungal Shannon diversity index (1.39415, 95% CI 1.0959 to 1.6924, *p* < 0.0001). While a decrease in the abundance of *Staphylococcus* was observed, this decrease was evident in all three groups and did not result in a significant treatment effect compared to the placebo for omiganan (2.53%, 95% CI −9.73 to 14.79, *p* = 0.6783) or ketoconazole (−7.46%, 95% CI −20.09 to 5.17, *p* = 0.2390). Concurrently, the bacterial biodiversity index did not significantly increase compared to the placebo in the omiganan treatment group (0.0922, 95% CI −0.1583 to 0.3427, *p* = 0.4621). However, an increased microbial biodiversity was observed in the ketoconazole group (0.3412, 95% CI 0.0859 to 0.5964, *p* = 0.0100). Overall, a strong correlation between the biodiversity index and the abundance of *Malassezia*, but not *Staphylococcus*, was observed (Figure S4). Full microbial profiles and an overview of *Malassezia* species detected using contact plates are presented in Figure S4 and Table S1, respectively.

## 3. Discussion

The use of topical AMPs holds promise for the treatment of SD since both antifungal and antibacterial involvement has been previously established in this disease. In this randomized, comparator- and placebo-controlled clinical trial, the efficacy of 1.75% omiganan gel was compared to its vehicle and standard-of-care 2% ketoconazole cream in a cohort of 36 patients with facial SD. Twice daily administration of omiganan did not result in significantly decreased clinical scores compared to the placebo. In contrast, a significant decrease in disease activity compared to the placebo was observed in the ketoconazole-treated group, which was paralleled by an improved barrier function and a reduced abundance of *Malassezia*. 

Multiple physician-reported scores were used to establish a treatment effect that yielded conclusive evidence of disease improvement in the ketoconazole-treated group versus the placebo. Clinical photography and OCT were performed to objectively reinforce these outcomes, but no differences were observed, even though these methods could detect a reduction of cutaneous inflammation over time in previous studies [25,26]. The inclusion of mild patients with a low baseline severity and a relatively small treatment effect size could have obscured these differences. Additionally, these localized digital assessments do not take a decreasing BSA into account, in contrast to the SDASI. Patient-reported outcomes showed improvement in all three treatment groups, highlighting the degree of placebo responses associated with metrics such as itch [27]. While the localized nature of SD impacted the responsiveness of the 5-D Itch score [28], all scores remained in agreement, as previously reported [29,30]. 

The absence of significant clinical effects in the omiganan-treated group was unexpected considering the clear microbiome-modulating effects found in vitro and in vivo [16,17,18,19,20,21,22,23,24]. Further investigation of the microbial composition showed that twice daily dosing with 1.75% omiganan did not significantly affect the abundance of *Staphylococcus* in our cohort of SD patients. Previous studies in atopic dermatitis patients demonstrated a clear staphylocidal effect and decreased microbial dysbiosis compared to the placebo with doses as low as 1% once or twice daily [23,24]. Involvement of *Staphylococcus*, and especially *S. aureus*, in the pathophysiology of atopic dermatitis has gained attention [6,7], and microbial profiling studies in SD have shown increased *Staphylococcus* abundances, accordingly [5,31,32,33]. However, this might not necessarily constitute dysbiosis as these studies have shown similar [32,33] or higher [31] biodiversity indexes compared to healthy controls. Moreover, this study was limited by insufficient phylogenetic resolution, which does not enable differentiation between *S. aureus* and *S. epidermidis* that are regarded as pathobiont and commensal species, respectively [34]. Ultimately, we demonstrated a decreased *Staphylococcus* abundance compared to baseline that did not correlate with clinical improvement, as all three treatment groups, including the placebo, showed a reduction in *Staphylococcus*. Combined with the fact that omiganan successfully alleviated dysbiosis at lower dosages, we postulate that microbial dysbiosis in SD may play a less important role based on the absence of a significant treatment-related effect.

While bacterial involvement might not be integral to SD pathophysiology, the involvement of *Malassezia* has been well established [1]. However, antifungal properties previously attributed to omiganan were not observed in this study [17,18,19]. Discord between the in vitro susceptibility of *Malassezia* and the clinical outcome of antimicrobial treatment has been described before and might in part be caused by the high between- and within-species variation present among different *Malassezia* strains [35]. One could hypothesize that the lipid-rich environment at seborrheic skin sites may have presented a mismatch for the aqueous glycerol-based formulation of omiganan, possibly hampering direct interaction with *Malassezia*. Abundant interaction might be important since omiganan is thought to exert its antimicrobial function by association with the cell wall through cationic interactions, thereby destabilizing it and causing cell death [36]. However, the precise mechanism of omiganan and derivatives might extend beyond the cell wall interactions [37,38]. Additionally, in vitro assays do not incorporate a host compartment, which could further distort translation to patients as omiganan has previously been shown to enhance innate immune responses [39]. However, the clinical scores used to establish the disease severity did not indicate an increased degree of inflammation upon omiganan treatment in this study nor in previous studies in atopic dermatitis [23,24].

In contrast to omiganan, ketoconazole demonstrated potent fungicidal effects. It remains debated whether ketoconazole’s therapeutic effect can be contributed solely to its ability to impair mycobial cell wall synthesis [40]. Ketoconazole has been shown to concomitantly reduce disease burden and *Malassezia* abundances in SD [41,42], but has also been efficacious without affecting *Malassezia* levels, sparking a debate on whether secondary interactions on microbial gene expression or host inflammatory responses are responsible [43]. In this study, we showed that treatment with ketoconazole reduced the disease severity and increased the mycobial diversity, which was strongly associated with a reduced abundance of *Malassezia*.

Interestingly, the analysis of skin permeability and underlying molecular ceramide markers of barrier integrity yielded significant differences for ketoconazole compared to the placebo. Barrier impairment is a hallmark of atopic dermatitis, and barrier recovery is associated with clinical improvement [44,45]. Similarly, barrier dysfunction is implicated in SD [46,47,48]. While ketoconazole significantly recovered barrier function compared to the placebo, the effects on the relative ceramide composition were small. Ceramides constitute part of the lipid matrix in the stratum corneum and are linked to skin barrier performance [49]. In this study, we observed a significant decrease in the abundance of Cer[NSc34] in the ketoconazole group. This, and the non-significant increase observed in the Cer[NS]:Cer[NP] ratio, increased the ceramide chain length and reduced the fraction of unsaturated Cer[NS] compared to baseline, can be associated with barrier recovery in atopic dermatitis and psoriasis [45,50,51,52,53,54,55]. Indeed, incorporation of more Cer[NS] [56,57], unsaturated lipids [58], and shorter ceramides [59] resulted in increased permeability of skin lipid models. While the individual parameters did not show significant changes, their joint contribution might amount to a significant synergistic effect on cutaneous barrier function. The combination of barrier recovery and amelioration of barrier markers in response to treatment underlines the involvement of barrier dysfunction in SD [46].

In conclusion, this randomized controlled study showed that topical administration of the AMP omiganan was safe and well tolerated but did not lead to clinical improvement in patients with facial SD. Microbial abundances and skin barrier parameters were not significantly altered compared to the placebo, either. In contrast, clinical scores significantly decreased in the ketoconazole group along with a potent fungicidal effect and an improved skin barrier function. This study underlines the efficacy of ketoconazole for the treatment of SD. Additionally, our comprehensive overview of the treatment response of ketoconazole as a comparative treatment supports the idea that *Malassezia* plays a central role in SD pathophysiology. Moreover, it demonstrated the dynamics of the skin barrier function in response to treatment, which supports previous studies that have implicated barrier dysfunction as a key factor in the pathophysiology of SD. 

## 4. Materials and Methods

### 4.1. Materials

Chloroform (Honeywell, Charlotte, NC, USA), methanol (Biosolve, Valkenswaard, The Netherlands), heptane (LiChorSolv, Merck, Darmstadt, Germany), UPLC-grade isopropyl alcohol (Biosolve, Valkenswaard, The Netherlands), and ethanol (Biosolve, Valkenswaard, The Netherlands) were of HPLC grade or higher. Reagent-grade potassium chloride (Sigma Aldrich, St. Louis, MO, USA) and ultrapure water from a Milli-Q Advantage A10 system (Merck, Darmstadt, Germany) were used. Synthetic ceramides and deuterated standards were purchased from Avanti Polar Lipids (Alabaster, AL, USA) or provided by Evonik (Essen, Germany).

### 4.2. Study Design, Randomization, and Treatments

The trial was registered under ClinicalTrials.gov Identifier NCT03688971 and EudraCT number 2017-003106-41. The study was performed from November 2019 to January 2022 at the Centre for Human Drug Research (Leiden, The Netherlands) following the Declaration of Helsinki principles and after obtaining ethical approval from the Stichting Beoordeling Ethiek Biomedisch Onderzoek (Assen, The Netherlands). Patients provided written informed consent prior to participation in the study. This was an evaluator- and subject-blinded parallel design evaluating the synthetic indolicidin analog, omiganan pentahydrochloride, a 12-amino-acid cationic peptide of H-Ile-Leu-Arg-Trp-Pro-Trp-Trp-Pro-Trp-Arg-Arg-Lys-NH_2_·5HCl, formulated in a glycerol-based gel (see protocol). Eligible patients were randomized to twice daily topical administration of 1.75% omiganan gel, vehicle gel, or 2.00% ketoconazole cream on all facial lesion sites for 28 days. The first administration was performed under the supervision of a dedicated and study-independent physician who also weighed the drug to monitor compliance and exposure. All subjects and other study staff remained blinded. An independent statistician generated the randomization into blocks of 3. Patient visits were scheduled at baseline, 7, 14, 21, 28 (end of treatment, EOT), 35, and 42 (end of study, EOS) days. Washing the face or applying medication was prohibited from 12 h preceding a visit. Different skin sites were selected and monitored to accommodate all assessments. For each assessment, the same site was monitored on all visits. The full study protocol and assessment schedule are provided in the Supplementary Materials. 

### 4.3. Patients

Included patients exhibited mild-to-moderate facial SD, as defined by an Investigator’s Global Assessment (IGA) score of ≤2 at screening, without any other clinically significant conditions, recent tanning, or recent exposure to other SD treatments. Recent exposure was defined as two weeks for topical treatments and anti-dandruff shampoo, three weeks for phototherapy, and four weeks for systemic treatment. Diagnosis of SD was confirmed by a dermatologist. 

### 4.4. Physician- and Patient-Reported Scoring

SD severity was scored by the Seborrheic Dermatitis and Severity Index (SDASI) [60], limited to only the facial extent, 5-point IGA (0, clear; 1, almost clear; 2, mild; 3, moderate; 4, severe), and the percentage of affected body surface area (%BSA). Patients completed the Dermatology Life Quality Index (DLQI) [61] and the 5-Domain Itch score [28] in-clinic and self-reported the daily 0–100 Numeric Rating Scale (NRS) Itch. 

### 4.5. Cutaneous Imaging

Cross-polarized facial photography was performed using a VISIA-CR (Canfield Scientific, Parsippany-Troy Hills, NJ, USA). The erythema index was determined through ImageJ (version 1.51 h CITE) based on Yamamoto et al. [62]. An original red, green, and blue (RGB) image was split, and the red and green channels were log-transformed. Subsequently, the green image was subtracted from the red channel and the resulting image was multiplied by 3. The erythema index was determined from a subject-specific region of interest that spanned 380,000 pixels. Epidermal thickness, superficial roughness, and the average degree of vascularization between 0.1 and 0.25 mm were determined by optical coherence tomography using a Vivosight Dx OCT and proprietary VivoTools 4.12 software (Michelson Diagnostics, Kent, UK). 

### 4.6. Trans-Epidermal Water Loss Measurements

Trans-epidermal water loss (TEWL) was determined using an AquaFlux AF200 (Biox Systems Ltd., London, UK) after the subjects acclimatized to the controlled environmental conditions (humidity < 60%, temperature 22 ± 2 °C) for at least 15 min. TEWL was normalized with an additional measurement of non-lesion skin elsewhere on the face to account for between-day variation, as described before [24]. 

### 4.7. Lipidomics Analysis Using Sebum Measurements and Liquid Chromatography-Mass Spectrometry after Tape-Stripping

Sebum levels were measured in triplicate on adjacent skin using a Sebumeter SM815 (Courage and Khazaka, Cologne, Germany). The average of three measurements was calculated. 

For lipidomics, the stratum corneum was sampled with 5 subsequent polyphenylene sulfide tape strips (Nichiban, Tokyo, Japan), of which the first one was discarded. Pressure was applied to each tape with a D500 D-squame Pressure Instrument (CuDerm Corporation, Dallas, TX, USA). A 16 mm-diameter hole was punched out from the section that was pressed onto the skin, and the tape was stored in chloroform:methanol (2:1). For extraction, tapes were shaken at 40 °C for one hour each in chloroform:methanol (2:1), chloroform:methanol:water (1:2:0.5), chloroform:methanol (1:1), and heptane:isopropylalcohol (1:1). The solvent was collected, and a liquid–liquid extraction was performed with the addition of 0.25 M potassium chloride. The organic layer was washed with chloroform, filtered with 0.45 µm PVDF syringe filters (Grace, Deerfield, IL, USA), and concentrated. Analysis was performed as described by Boiten et al. [63]. Samples were dried and reconstituted in heptane:chloroform:methanol (95:2.5:2.5) containing 10 µM of CER[N(24 deu)S(18)] at a concentration of 20 tapes/mL. Separation was achieved on an Acquity UPLC H-class system (Waters, Milford, MA, USA) with a normal-phase PVA-silica column (5 μm particles, 100 × 2.1 mm i.d.) (YMC, Kyoto, Japan), with a binary gradient between heptane and heptene:isopropylalcohol:ethanol (2:1:1) from 98:2 to 50:50 at a flow rate of 0.8 mL/min. An XEVO TQ-S mass spectrometer (Waters, Milford, MA, USA) with APCI in positive ion mode, scanning from 350 to 1200 *m/z*, was used. Quality control samples from combined stratum corneum extracts and standard calibration curves containing 50, 20, 10, 5, 2, 1, 0.5, and 0 μM of several ceramides (Cer[NS, NdS, NP, AS, EOS, and EOP]) in triplicate were added to the run. All detectable ceramides from the following ceramide classes were integrated based on their monoisotopic mass using TargetLynx V4.1 (Waters, Milford, MA, USA): Cer[NdS], Cer[NS], Cer[NP], Cer[NH], Cer[AdS], Cer[AS], Cer[AP], Cer[AH], Cer[OdS], Cer[OS], Cer[OP], Cer[OH], Cer[EOdS], Cer[EOS], Cer[EOP], and Cer[EOH]. The area under the curve (AUC) values of the monoisotopic masses were corrected for the internal standard in Excel (Microsoft 365, Redmond, WA, USA). The monoisotopic AUC was further corrected by the degree of water loss, theoretic 13C isotope distribution, and differences in ionization at higher molecular masses. The response per ceramide was converted to relative data using the total corrected AUC, and calculations were performed after grouping individual ceramides by their aforementioned class for further graphing. The Cer[NS]:Cer[NP] ratio is the ratio between the relative total abundance of the subclasses. Average ceramide chain length was derived from the non-acyl ceramides moiety: Cer[NdS], Cer[NS], Cer[NP], Cer[NH], Cer[AdS], Cer[AS], Cer[AP], and Cer[AH]. The abundance of Cer[NSc34] and the amount of unsaturated Cer[NS] were determined relative to all Cer[NS] detected. Ceramide nomenclature from Motta et al. was used (Figure S1) [64].

### 4.8. Microbial Profiling

Skin was swabbed for 10 s while constantly rotating using a sterile polyester-tipped applicator (Puritan, Guilford, ME, USA) soaked in 0.9% NaCl. Swabs were stored in DNA/RNA shield lysis buffer and beat beads (Zymo Research, Irvine, CA, USA) at −80 °C until analysis. Extraction, sequencing, and data generation were performed at Baseclear B.V. (Leiden, The Netherlands). Extraction was performed using a ZymoBIOMICS DNA Miniprep Kit (Zymo Research) according to the manufacturer’s instructions. The sample was split, and 16s RNA region v3–v4 or internal transcribed spacer region 2 (ITS2) sequencing was performed on a NovaSeq 6000 or MiSeq (Illumina, San Diego, CA, USA) after the appropriate sample quality control, for bacterial and fungal profiling, respectively. The reads were classified using the RDP database for bacterial [65] and the UNITE ITS gene database for fungal [66] classification and extracted from the Genome Explorer portal (Baseclear B.V., Leiden, The Netherlands), before genera contributing < 1% of the total hits were excluded using Python scripts (version 3.8.0, Python Software Foundation, Wilmington, DE, USA) and the relative abundance of the remaining microbes was determined. The relative abundance of genera comprising > 1% of the total hits were included in the microbial profile (Figure S5). 

### 4.9. Malassezia Species Identification by Matrix-Assisted Laser Desorption Ionization–Time of Flight Mass Spectrometry

Agar plates of 5.5 cm in diameter with modified Dixon agar medium (Tritium Microbiology B.V., Eindhoven, The Netherlands) were pressed against the skin for 20 min. Plates were transferred to the Alrijne Hospital (Leiden, The Netherlands) and cultured for up to 21 days at 33 °C. If mycological growth was observed, an isolate was taken and frozen in microbank 2D tubes (pro-lab diagnostics, Richmond Hill, ON, Canada). Isolates were transported to the Westerdijk Fungal Biodiversity Institute (Utrecht, The Netherlands), defrosted, and further cultured on modified Leeming and Notman medium before analysis using matrix-assisted laser desorption ionization–time of flight mass spectrometry, as described by Kolecka et al. [67]. 

### 4.10. Statistical Analysis

Calculations were performed using SAS version 9.4 (SAS Institute, Cary, NC, USA). Group size was based on a previous trial with omiganan with significant clinical effects rather than a formal power calculation [24]. A mixed model of repeated measures, with treatment, time, and treatment-by-time as fixed factors, and time as a repeated factor within patients, as well as an unstructured variance–covariance matrix were used. Contrasts between the omiganan and ketoconazole versus the placebo group were reported for the EOT with *p*-values as: * *p* ≤ 0.05, ** *p* ≤ 0.01, and *** *p* ≤ 0.001. Graphs show the change from the baseline least square means estimates and the 95% confidence interval (CI).

## Figures and Tables

**Table 1:** Demographics of the study population and adverse events.

	Omiganan 1.75% (n = 12)	Ketoconazole 2% (n = 13)	Placebo (n = 12)
**Demographics**			
Sex, n (%)			
Female	1 (8.3%)	2 (15.4%)	2 (16.7%)
Male	11 (91.7%)	11 (84.6%)	10 (83.3%)
Age, mean (SD)	32.2 (11.0)	39.7 (20.7)	41.3 (12.5)
Race, n (%)			
Asian	0 (0%)	1 (7.7%)	0 (0%)
Mixed	0 (0%)	1 (7.7%)	0 (0%)
Other	1 (8.3%)	0 (0%)	0 (0%)
White	11 (91.7%)	11 (84.6%)	12 (100%)
**Exposure**			
Total dose (g), mean (SD)	18.83 (10.83)	18.38 (13.64)	21.35 (8.50)
Dose per day (mg), mean (SD)	716.48 (413.46)	726.78 (461.27)	896.37 (349.10)
Dose per application (mg), mean (SD)	402.77 (237.05)	402.82 (248.930)	479.32 (187.16)
**Treatment emergent adverse events**			
Total events	4	4	4
General disorders and administration site conditions			
Application site discomfort	1		
Influenza-like illness	1	1	
Infections and infestations			
Rhinolaryngitis			1
Injury, poisoning, and procedural complications			
Application site pruritus			1
Arthropod bite	1		
Ligament sprain		1	
Nervous system disorders			
Headache			2
Respiratory, thoracic, and mediastinal disorders			
Cough		1	
Rhinorrhea	1		

## Data Availability

The data that support the findings of this study are available from the corresponding author and/or trial sponsor upon reasonable request.

## Supplementary Materials

The following supporting information can be downloaded at: https://www.mdpi.com/article/10.3390/ijms241814315/s1.
