# Supplementary material for: Treatment with the Topical Antimicrobial Peptide Omiganan in Mild-to-Moderate Facial Seborrheic Dermatitis versus Ketoconazole and Placebo: Results of a Randomized Controlled Proof-of-Concept Trial
Source: Int J Mol Sci. 2023 Sep 20;24(18):14315. doi: 10.3390/ijms241814315 (PMC10531869; doi:10.3390/ijms241814315)
Supplement: Supplementary file 1 [file ijms-24-14315-s001.zip › ijms-2604495-supplementary.pdf]

Supplemental information pertaining to:

Topical ketoconazole but not the antimicrobial peptide omiganan is effective for the treatment mild-to-moderate facial seborrheic dermatitis compared to placebo and in a phase 2 randomized controlled trial.

J. Rousel, et al. (2023)

Supplemental methods

Please note that additional information is described in the study protocol available in the supporting information online.

Image processing for erythema assessments

The erythema index was determined from based on the method by Yamamoto, et al. (2008)[1] using ImageJ (version 1.51h)[2]. An original Red Green Blue (RGB) image was split and the Red and Green channels log transformed. Subsequently, the green image was subtracted from the red channel and the resulting image multiplied by 3. The erythema index was determined from a subject specific region of interest that spanned 380000 pixels.

Microbial profiling by sequencing

The skin was swabbed for 10 seconds while constantly rotating using a sterile polyester tipped applicator (Puritan, Guilford, Maine, United States) soaked in 0.9% NaCl. Swabs were stored in DNA/RNA shield lysis buffer and beat beads (Zymo Research, Irvine, California, Unites States) at -80 °C until analysis. Extraction, sequencing and data generation was performed at Baseclear B.V. (Leiden, the Netherlands). Extraction was performed using a ZymoBIOMICS DNA Miniprep Kit (Zymo Research) according to manufacturer's instructions. The sample was split and 16s RNA region v3-v4 or Internal transcribed spacer region 2 (ITS2) sequencing was performed on a NovaSeq 6000 or MiSeq (Illumina, San Diego, California, United States of America) after appropriate sample quality control, for bacterial[3] and fungal profiling respectively. The Reads were classified using the RDP database for bacterial[3] and UNITE ITS gene database for fungal[4] classification and extracted from the Genome Explorer portal (Baseclear B.V., Leiden, the Netherlands) before genera contributing <1% of the total hits were excluded using Python scripts (version 3.8.0, Python Software Foundation, Wilmington, Delaware, United States) and the relative abundance of the remaining microbes was determined.

Malassezia species identification by matrix-assisted laser desorption ionization-time of flight mass spectrometry

5.5 cm agar plates with modified Dixon agar medium (tritium microbiology B.V., Eindhoven, the Netherlands) were pressed against the skin for 20 minutes. Plates were transferred to the Alrijne Hospital (Leiden, the Netherlands) and cultured for up to 21 days at 33 °C. If mycological growth was observed, an isolate was taken and frozen in microbank 2D tubes (pro-lab diagnostics, Richmond Hill, ontario, Canada). Isolates were transported to the Westerdijk Fungal Biodiversity Institute (Utrecht, the Netherlands), defrosted and further cultured on modified Leeming and Notman medium before analysis using matrix-assisted laser desorption ionization-time of flight mass spectrometry as described earlier[5].

Tape stripping and lipidomics with liquid chromatography-mass spectrometry

Chloroform (Honeywell, Charlotte, North Carolina, United States), methanol (Biosolve, Valkenswaard, the Netherlands), heptane (LiChorSolv, Merck, Darmstadt, Germany), UPLC grade isopropyl alcohol (Biosolve, Valkenswaard, the Netherlands) and ethanol (Biosolve, Valkenswaard, the Netherlands) were of HPLC grade or higher. Reagent grade potassium chloride (Sigma Aldrich, Saint-Louis, Missouri, USA) and ultrapure water from a Milli-Q Advantage A10 system (Merck, Darmstadt, Germany) were used. Synthetic ceramides and deuterated standards were purchased from Avanti Polar Lipids (Alabaster, Alabama, United States) or provided by Evonik (Essen, Germany). Ceramide lipidomics was performed as described by Boiten et al. (2016)[6]. Stratum corneum was sampled with 5 subsequent polyphenylene sulfide tape strips (Nichiban, Tokyo, Japan) of which the first one was discarded. Pressure was applied to each tape with a D500 D-squame Pressure Instrument (CuDerm Corporation, Dallas, TX, United States). A 16mm diameter hole was punched out from the section that was pressed onto the skin and the tape stored in chloroform:methanol (2:1). For extraction, tapes were shaken at 40 °C for one hour each in chloroform:methanol (2:1), chloroform:methanol:water (1:2:0.5), chloroform:methanol (1:1) and heptane:isopropylalcohol (1:1). The solvent was collected and a liquid-liquid extraction performed with the addition of 0.25M potassium chloride. The organic layer was washed with chloroform, filtered with 0.45 µm PVDF syringe filters (Grace, Deerfield IL, USA) and concentrated. For analysis, samples were dried and constituted in heptane:chloroform:methanol (95:2.5:2.5) containing 10 µM CER[N(24deu)S(18)]. Separation was achieved on an Acquity UPLC H-class (Waters, Milford, MA, USA) with a normal phase PVA-silica column (5 µm particles, 100 × 2.1 mm i.d.) (YMC, Kyoto, Japan) with a binary gradient between heptane and heptene:isopropylalcohol:ethanol (2:1:1) from 98:2 to 50:50at a flow rate of 0.8 ml/min. An XEVO TQ-S mass spectrometer (Waters, Milford, MA, USA) with APCI in positive ion mode scanning from 350 to 1200 m/z was used. Quality control samples from combined stratum corneum extracts and standard calibration curves containing 50, 20, 10, 5, 2, 1, 0.5, 0 µM of several ceramides (Cer[NS, NdS, NP, AS, EOS and EOP] in triplicate were added to the run. All detectable ceramides from the following ceramide classes were integrated based on their monoisotopic mass using TargetLynx V4.1 (Waters, Milford, MA, USA); Cer[NdS], Cer[NS], Cer[NP], Cer[NH], Cer[AdS], Cer[AS], Cer[AP], Cer[AH], Cer[OdS], Cer[OS], Cer[OP], Cer[OH], Cer[EOdS], Cer[EOS], Cer[EOP], Cer[EOH]. Area Under the Curve (AUC) of the monoisotopic masses were corrected for the internal standard in Excel (Microsoft 365, Redmond, Washington, United States). The monoisotopic AUC was further corrected by the degree of water loss, theoretic 13C isotope distribution and differences in ionization at higher molecular masses. The response per ceramide was converted to relative data using the total corrected AUC and calculations were made after grouping individual ceramides by their aforementioned class further graphing. The Cer[NS]:Cer[NP] ratio is the ratio between the relative total abundance of the subclasses. Average ceramide chain length is derived from the non-acyl ceramides moiety: Cer[NdS], Cer[NS], Cer[NP], Cer[NH], Cer[AdS], Cer[AS], Cer[AP], Cer[AH]. The abundance of Cer[NSc34] and the amount of unsaturated Cer[NS] is determined relative to all Cer[NS] detected.

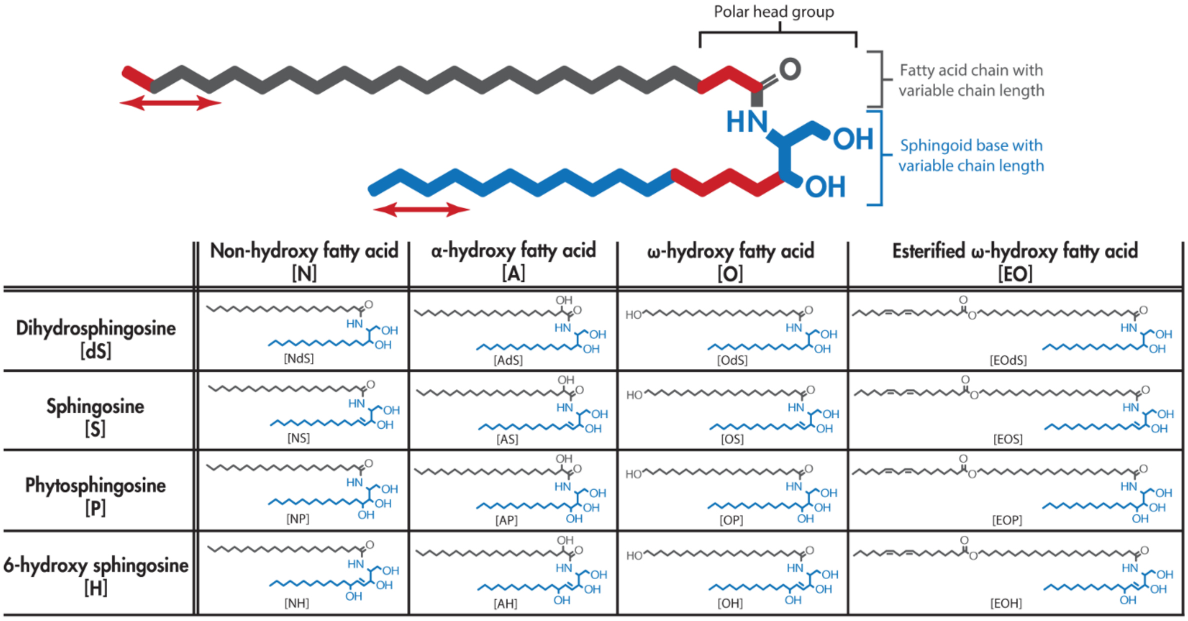

Supplemental figure S1: General structure of a ceramides. Differences in the ceramide headgroup architecture is indicated using the naming convention conceived by Motta *et al.* (1993). Ceramides are composed of a fatty acid tail denoted by N, A or EO and a sphingoid base denoted by -dS, S, P or H. The carbon chains attached to the polar head group can vary in length. This image was adapted from Janssens *et al.* (2012)[7].

CONSORT
TRANSPARENT REPORTING of TRIALS

CONSORT 2010 Flow Diagram

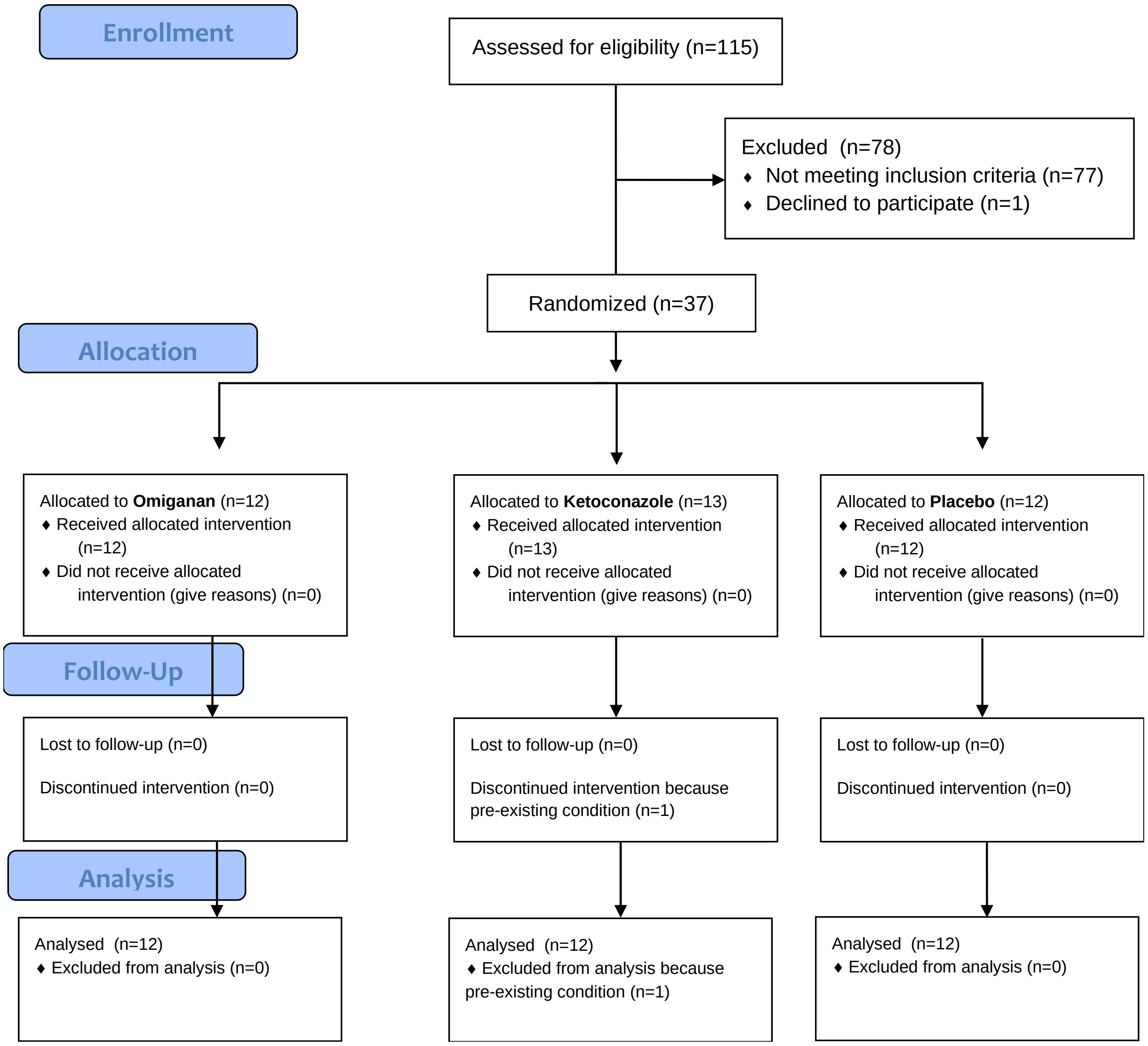

Supplemental figure s2: Consort 2010 flow diagram.

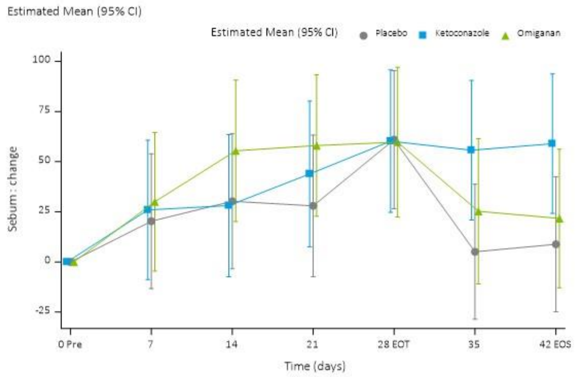

Supplemental figure s3: Change in the superficial sebum levels over time. Data shown represents the mean and 95% Confidence interval (CI). No significant change is observed compared to placebo for omiganan (2.53, 95% CI -9.73 to 14.79, p = 0.678) and ketoconazole (-7.46, 95% CI -20.09 to 5.17, p = 0.239).

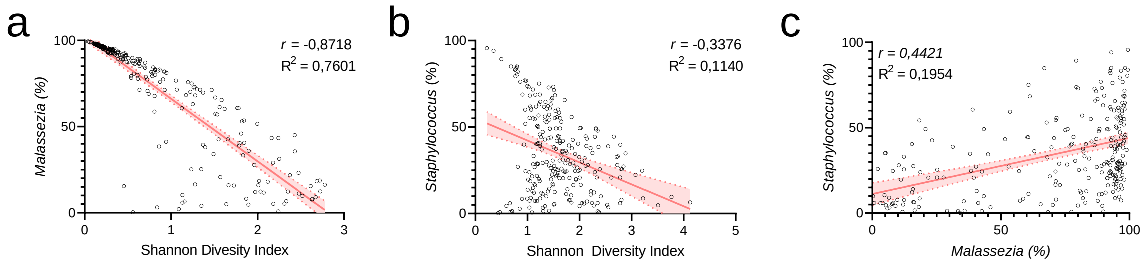

Supplemental figure s4: Correlation between the fungal Shannon diversity index and *Malassezia* abundance (A), between the bacterial Shannon diversity index and *Staphylococcus* abundance (B) and between the abundances of *Malassezia* and *Staphylococcus* (C). Datapoints from all samples throughout the study have been plotted. A straight line with least square fit and 95% confidence area is plotted through the data. Pearson correlation coefficient and goodness of fit of the trendline is shown.

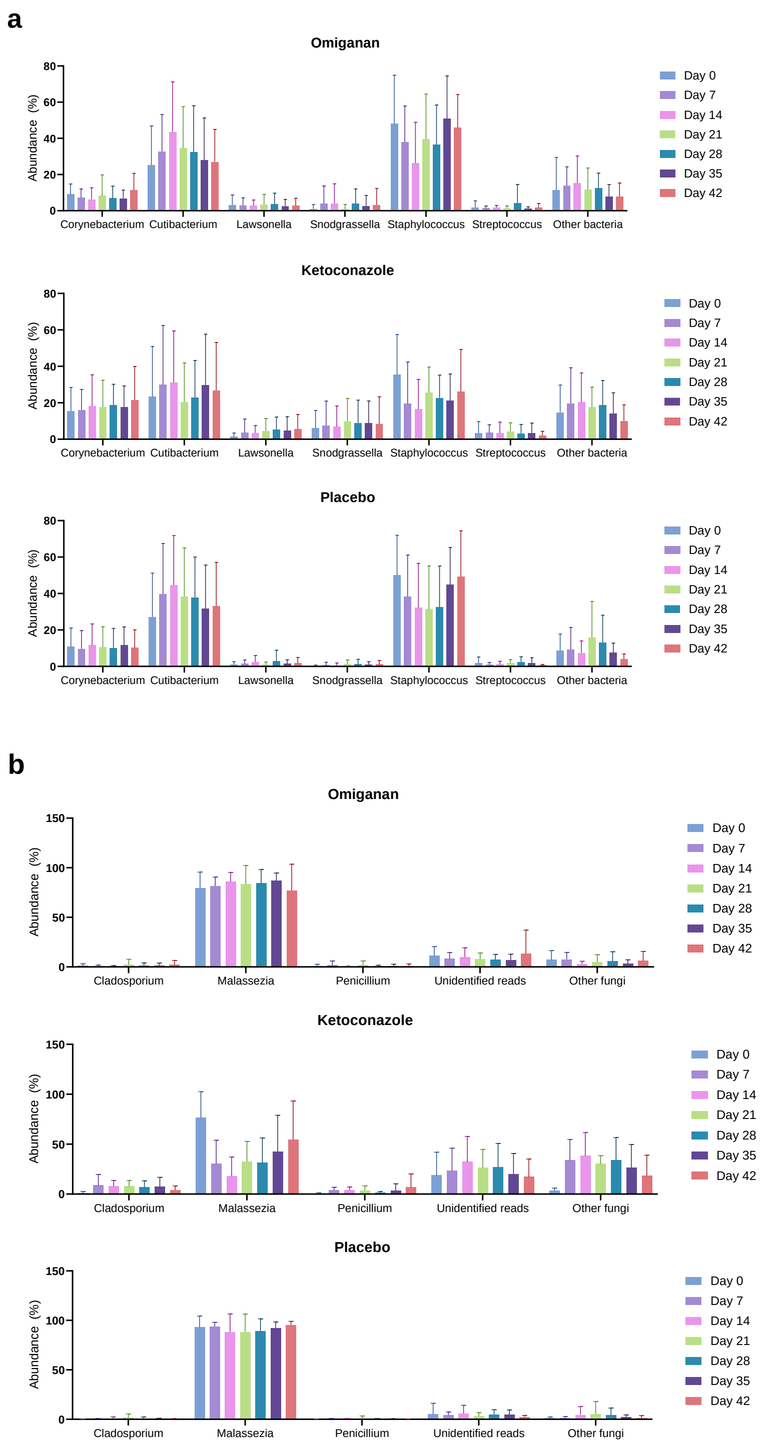

Supplemental figure s5: The bacterial (a) and fungal (b) profile over time per treatment group. Error bars represent standard deviations. Bacteria that do not comprise 1% of the total abundance found between samples are grouped under “other”.

Supplemental table s1: Counts of *Malassezia* species identified by matrix-assisted laser desorption ionization-time of flight mass spectrometry per treatment. No agar plate yielded more than 1 different species of *Malassezia*. The total represents the total amount of detected species identified from the total of 12 agar plates.

	Day 0	Day 7	Day 14	Day 21	Day 28	Day 35	Day 42
Omiganan							
M. slooffiae	1	0	0	0	1	1	0
M. sympodialis	2	2	4	1	2	2	1
M. globosa	1	2	0	3	0	1	3
M. restricta	1	0	0	0	1	0	0
M. obtusa	0	0	0	0	1	0	0
Total	5/12	4/12	4/12	4/12	5/12	4/12	4/12
ketoconazole							
M. slooffiae	3	0	0	0	0	0	0
M. sympodialis	2	0	0	0	0	1	0
M. globosa	1	0	0	0	0	0	0
M. restricta	0	0	0	0	0	0	0
M. obtusa	0	0	0	0	0	0	0
Total	6/12	0/12	0/12	0/12	0/12	1/12	0/12
Placebo							
M. slooffiae	1	1	0	0	1	0	1
M. sympodialis	3	1	2	0	3	1	1
M. globosa	2	0	0	3	0	0	0
M. restricta	1	1	0	0	1	0	0
M. obtusa	0	0	0	0	0	0	0
Total	7/12	3/12	2/12	3/12	5/12	1/12	2/12
